# Integration of Tobacco Treatment Services into Cancer Care at Stanford

**DOI:** 10.3390/ijerph17062101

**Published:** 2020-03-22

**Authors:** Kathleen Gali, Brittany Pike, Matthew S. Kendra, Cindy Tran, Priya Fielding-Singh, Kayla Jimenez, Rachelle Mirkin, Judith J. Prochaska

**Affiliations:** 1 Stanford Prevention Research Center, Department of Medicine, School of Medicine, Stanford University, Stanford, CA 94305, USA; kgali@stanford.edu (K.G.); bpike@stanfordhealthcare.org (B.P.); priyafs@stanford.edu (P.F.-S.); 2 Stanford Health Care, Stanford, CA 94305, USA; cindytran@stanfordhealthcare.org (C.T.); rmirkin@stanfordhealthcare.org (R.M.); 3 Department of Psychiatry and Behavioral Sciences, School of Medicine, Stanford University, Stanford, CA 94305, USA; mkendra@stanford.edu; 4 PGSP-Stanford Psy.D. Consortium, Palo Alto University, Palo Alto, CA 94304, USA; kaylaj@stanford.edu

**Keywords:** smoking cessation, tobacco treatment, cancer care, quality improvement, oncology

## Abstract

As part of a National Cancer Institute Moonshot P30 Supplement, the Stanford Cancer Center piloted and integrated tobacco treatment into cancer care. This quality improvement (QI) project reports on the process from initial pilot to adoption within 14 clinics. The Head and Neck Oncology Clinic was engaged first in January 2019 as a pilot site given staff receptivity, elevated smoking prevalence, and a high tobacco screening rate (95%) yet low levels of tobacco cessation treatment referrals (<10%) and patient engagement (<1% of smokers treated). To improve referrals and engagement, system changes included an automated “opt-out” referral process and provision of tobacco cessation treatment as a covered benefit with flexible delivery options that included phone and telemedicine. Screening rates increased to 99%, referrals to 100%, 74% of patients were reached by counselors, and 33% of those reached engaged in treatment. Patient-reported abstinence from all tobacco products at 6-month follow-up is 20%. In July 2019, two additional oncology clinics were added. In December 2019, less than one year from initiating the QI pilot, with demonstrated feasibility, acceptability, and efficacy, the tobacco treatment services were integrated into 14 clinics at Stanford Cancer Center.

## 1. Introduction

Approximately 30% of cancer deaths in the U.S. are attributed to smoking [1]. Continued smoking after a cancer diagnosis can complicate cancer treatments by interacting with medications, increasing the risk of treatment side-effects, and decreasing wound healing time [2,3,4,5]. Quitting smoking as early as possible after a cancer diagnosis not only eliminates these complications but also has been shown to increase survival and well-being while decreasing the risks of disease recurrence and second primary tumors [6,7]. For these reasons, tobacco cessation services are recommended as a standard component of cancer treatment [8].

Patients diagnosed with cancer who smoke often want to quit but face challenges. Research indicates that about 50% of patients who smoked before a cancer diagnosis continued to smoke during treatment [9]. Among patients who continued to smoke after undergoing treatment for head and neck cancer, most reported being motivated to quit and making past attempts to quit, but few entered a formal tobacco treatment program or received pharmacotherapy to deal with nicotine withdrawal or dependence, and most returned to smoking within 1 month posttreatment [10]. 

The National Comprehensive Cancer Network’s (NCCN) guidelines for treating tobacco dependence in cancer care recommend repeated interventions (even as brief as 3 min) to encourage engagement in tobacco treatment, including counseling and the use of cessation medications [8,11]. Combination medication and behavioral therapy is considered more effective compared to behavioral therapy or medication alone. Food and Drug Administration (FDA)-approved medications are nicotine replacement therapy (NRT; patch, lozenge, gum, nasal spray, and inhaler), bupropion, and varenicline. Referral resources for quitting smoking are available through state quitlines and online, for example, the National Cancer Institute’s (NCI) smokefree.gov website [12].

Historically, clinical systems have used an “opt-in” approach to tobacco treatment, whereby patients have to request or accept a referral from their oncology providers. As an alternative, in an “opt-out” model, all patients who use tobacco are automatically referred for evidence-based tobacco cessation treatment, with the option to opt-out from engaging in care. The opt-out model has increased patient reach and engagement [13], and quit rates [14]. “Opt-out” models for tobacco treatment have proven feasible in inpatient and cancer care settings [15,16]. 

Despite the promotion of NCCN guidelines for effective tobacco cessation strategies, systemwide adoption of comprehensive tobacco treatment in cancer settings has been low. In a survey of NCI-designated comprehensive cancer centers, 40% reported not having any tobacco treatment available for patients [17]. System-level barriers to care include lack of organizational prioritization, staff training, clarity in roles and responsibilities, and resources for treating tobacco [17,18]. Research has examined oncologists’ practice patterns regarding tobacco assessment, cessation support, perceptions of tobacco use, and barriers to providing cessation support for patients with cancer. In a survey of 1101 members of the American Society of Clinical Oncology, 80% reported they always ask about tobacco use at the initial visit, 58% always advised those who use tobacco to quit, and 38% reported assessing tobacco at follow-up. Reported barriers to routinely providing cessation support were inadequate training (38%) and perceiving patients as resistant to tobacco treatment (74%) [19]. Asking about tobacco use and advising cessation are necessary first steps for treating tobacco addiction; however, ongoing support and combination treatment often are necessary to prevent relapse [20]. Insurance coverage for tobacco treatment in the U.S. also remains inconsistent and often non-comprehensive, creating access challenges for patients. While the Affordable Care Act and other federal laws require most health insurance plans to cover some tobacco treatment, policymakers may not enforce these requirements [21].

Despite the known harms of tobacco use and the recommended need for tobacco cessation treatment in the oncology setting, the clinical trial evidence is weak with regard to outcomes. A recent meta-analysis of 21 smoking cessation studies among cancer survivors reported a nonsignificant treatment effect [22]. Subgroup analyses of specific behavior change techniques (e.g., general encouragement and enhancing self-efficacy), found to be effective in other patient populations (e.g., patients with chronic obstructive pulmonary disease [COPD] and patients undergoing surgery), also were nonsignificant. Novel approaches appear warranted and particularly with consideration of the unique aspects of cancer care systems and cancer survivorship. More broadly, but with relevance here, there have been calls for “practice-based production” of evidence that maximizes external validity, conducted in clinical settings with real-world patient populations [23].

This quality improvement (QI) project aimed at integrating accessible, tobacco treatment, consistent with NCCN guidelines, into cancer care at a major cancer center. With novelty, the treatment approach combined an opt-out model to maximize outreach, a telemedicine-delivered treatment and virtual same-day delivery pharmacy to maximize reach, and a training program with individual counseling to maximize engagement. The planning effort by the Tobacco Treatment Service team started in August 2018 and has been supported by a Moonshot P30 Supplement Award (P30CA124435-11S1) as part of the National Cancer Institute’s Cancer Center Cessation Initiative (C3I). By adhering to the “Plan, Do, Study, Act” (PDSA) methodology [24,25], the team implements weekly improvements to advance the quality and effectiveness of services provided to patients and family members. Reported here is the process, methods, and results from engaging providers and patients in three pilot clinics at the Stanford Cancer Center Palo Alto location (Head and Neck Oncology, Thoracic Oncology, and GI Surgery Oncology) and the subsequent efforts for center-wide implementation. Project activities were reviewed by Stanford Medicine’s Institutional Review Board and determined to fall within the domain of a quality improvement evaluation (Protocol #48420). 

## 2. Materials and Methods 

The Stanford Cancer Center (SCC), an NCI-designated Comprehensive Cancer Center, is located in Northern California with sites in Palo Alto, Redwood City and San Jose, California. The catchment area encompasses nine counties: Alameda, Merced, Monterey, San Benito, San Mateo, Santa Clara, Santa Cruz, San Joaquin, and Stanislaus Counties. Serving a vast area, SCC’s annual number of newly registered cancer patients is over 6500. State surveillance data in 2018 for adults (18–80 years) in the SCC’s catchment area indicate a smoking prevalence of 6% for women and 15% for men (11% overall) [26].

The Stanford Tobacco Treatment Service was developed with the goal of connecting patients receiving cancer care and their family members who use tobacco to evidence-based, high-quality tobacco cessation treatment. While combustible cigarettes are the main tobacco product of use, patients using any form of tobacco (e.g., cigars, smokeless tobacco, e-cigarettes) are eligible for treatment. 

The Stanford Tobacco Treatment Service consisted initially of 1.3 supported full-time equivalent (FTE) team members, increased over time to 2.3 FTE, including a full-time (initially half-time) certified tobacco treatment specialist, two program managers at combined 1 FTE (initially 0.5 FTE), and three addiction medicine doctoral-level clinicians together contributing 0.3 FTE. The service also is an 8-hour a week practicum site for four psychology students, providing supervised patient care. Ancillary support to the program is provided by advanced practice providers and management staff in the oncology clinics, the SCC’s data/quality/IT improvement systems team, and the virtual pharmacy partner with pharmacists certified to prescribe NRT. 

Onboarding and ongoing training activities for team members include in-services (by the team’s addiction medicine clinicians and the California Smoker’s Helpline clinical director), key literature (e.g., NCCN guidelines, Surgeon General Reports, review articles), and webinars. Additionally, one staff member completed an accredited tobacco treatment specialist program and three team members participated in Memorial Sloan–Kettering’s Assessment and Treatment of Tobacco Dependence in Cancer Care multiday workshop. 

### 2.1. Pre-Implementation Assessment at a Pilot Clinic 

As part of this QI initiative, the Stanford Tobacco Treatment Service partnered first with Stanford’s Head and Neck Oncology Clinic as a pilot site to assess barriers and facilitators to cancer patients accessing tobacco treatment. The clinic was chosen for its elevated smoking prevalence, receptivity to the project, and >95% tobacco screening rate yet low rate of treatment referrals (<10%) and low engagement of patients in tobacco cessation treatment. Prior to the pilot, <1% of the clinic’s patients who reported smoking were engaging in tobacco cessation treatment. Tobacco quit success rates were not tracked during this timeframe. While the clinicians viewed tobacco dependence as medically relevant, the clinics were not resourced with tobacco treatment specialists and the oncology care providers’ time was limited. The clinicians and the clinic leadership expressed interest in a pilot partnership to connect identified tobacco users with flexible and convenient cessation treatment services.

Critically, the integrated model needed to fit within the clinicians’ preexisting workflow. As a first step, the Stanford Tobacco Treatment Service team conducted a gemba [27], which refers to the act of shadowing a team, workflow, and/or process in the actual place where the work is done and value is created. In this case, the team shadowed in clinic to learn about the tobacco-screening workflow and barriers to referring patients for treatment. The clinic was observed to follow an opt-in model, reliant on the clinicians to generate tobacco treatment referrals in the electronic health record (EHR). The system’s sole tobacco cessation program at the time was an in-person group offered one hour a week at the medical center’s psychiatry clinic. The reliance on clinician referrals, the location of the tobacco cessation group external to oncology, and the in-person format and limited frequency limited patient engagement. Insurance coverage also was a barrier to care, and stigma may have hindered engagement given that the tobacco treatment service was offered in a psychiatric setting. 

Through attendance at clinic meetings, the in-clinic shadowing, and conversations with clinic leadership, the tobacco treatment QI team developed strong relationships with medical assistants, nurses, providers, and administration to ensure the prioritization and sustainability of tobacco treatment integration. The identified barriers informed intervention development and implementation. Developed was a flexible menu of accessible tobacco treatment services to offer patients and their family members as a covered benefit throughout and beyond their oncology treatment.

### 2.2. Intervention Development and Implementation

An automated opt-out referral process was developed to ensure that all patients treated in the Head and Neck Oncology Clinic were screened for tobacco use and provided opportunities to engage in tobacco treatment. The QI workflow is described here. 

First, patients were screened for tobacco use at every clinic visit by the oncology team. Once identified by a medical assistant, a magnet was placed either on the exam room door or on the clinic white board to indicate a positive tobacco screen. These magnets were visible to all staff and providers. Oncology care providers gave patients identified as tobacco users a tobacco-treatment brochure and informed them that the Stanford Tobacco Treatment Service would call them within 1 week to discuss the services available. Patients were given the option to opt-out of being contacted. When a patient opted-out of treatment, the provider emailed the Stanford Tobacco Treatment Service team to not contact that patient. 

Next, the Stanford Tobacco Treatment Service generated a weekly report from the EHR with contact information for all patients positively screened for tobacco use in the clinic. A tobacco treatment team member called every patient on the report up to three times, leaving voicemails as needed. Additionally, messages were sent via the EHR patient portal introducing the tobacco treatment specialist and tobacco treatment options available to patients and family members for a free or reduced price. 

The menu of treatment services included counseling and/or cessation medications. Counseling was offered in-person at Stanford before or after their clinic visit, individually or via group, or virtually through telemedicine or over the phone. The cessation counseling was offered to patients by predoctoral clinical psychology students, supervised by the team’s licensed clinical psychologists. Family members also were eligible to receive counseling support and/or cessation medications. The counseling was offered as a three-session treatment with the option to continue. Translation services were available by phone through Stanford Health Care’s Translator Services. Patients who selected telephone-based counseling were referred to the toll-free California Quit Line through an electronic referral portal where a trained counselor reached out to patients over the phone to offer free, ongoing, telephone-based counseling in six languages. Information on NCI’s smokefree.gov web, chat, and phone-support services also was provided.

For cessation medication, patients were connected to Alto, a virtual pharmacy in Northern California, that offered same-day delivery of cessation medications at a discounted price. A partner in this QI initiative, Alto’s pharmacists furnished NRT following patient consultation and offered a two-week free trial to the patients and their family members. For patients interested in varenicline or bupropion, consultation via telemedicine with the Stanford Tobacco Treatment Service’s addiction medicine physician was available. Prescriptions for varenicline or bupropion were written by the addiction medicine physician and could be filled, often with same day delivery, by Alto.

To address insurance eligibility barriers, tobacco treatment was offered at no charge as a covered benefit for Stanford patients receiving cancer care. To streamline care delivery, with fewer hand-offs, services for tobacco treatment were offered through the Stanford Cancer Center. Housing the tobacco treatment services within the Stanford Cancer Center and framing the services as part of cancer care may minimize stigma. Patients were made aware of the new service through brochures and a treatment service website. 

Lastly, to communicate program metrics and to further refine service delivery, once per month, a Stanford Tobacco Treatment Service team member attended the clinic’s morning huddle and/or monthly staff meeting to present patient engagement data and to discuss any improvement opportunities with the screening process or service infrastructure.

### 2.3. Expansion

Six months after initiating the pilot in the Head and Neck Oncology Clinic, two additional clinics were engaged: Thoracic Oncology and GI Surgery Oncology. The Stanford Tobacco Treatment Service team initiated gembas in each clinic to learn their workflows and to identify the need for modifications to meet the unique needs of each clinic. Based on what was learned in Head and Neck Oncology, clear expectations were set with clinic leadership regarding the tobacco treatment team’s scope of work and time to outreach. Partnerships were developed with social work and case management to ensure patients with needs beyond tobacco dependence were supported appropriately. 

## 3. Results

Prior to the QI initiative, between January 2018 through the end of December 2018, a total of 5735 patients were seen at the three participating pilot clinics; 5580 (97%) patients were screened for tobacco use; 242 (4%) patients had current tobacco use indicated; and 42 (17%) patients had clinician tobacco treatment assistance documented. Clinician assistance to patients’ tobacco use included prescription for cessation medication (n = 7), brief provider counseling (n = 2), and/or a referral to the psychiatry clinic’s tobacco treatment group (n = 35). Data for the psychiatry clinic’s tobacco treatment group were available only for Q1 of 2018 and indicated that the service received 9 referrals from the three oncology clinics of interest; however, none of the referred patients completed an intake or engaged in treatment. Likely barriers were insurance, distance, scheduling, and care being based in psychiatry rather than within oncology. Tobacco quit success rates were not tracked during this timeframe.

**Figure 1:**
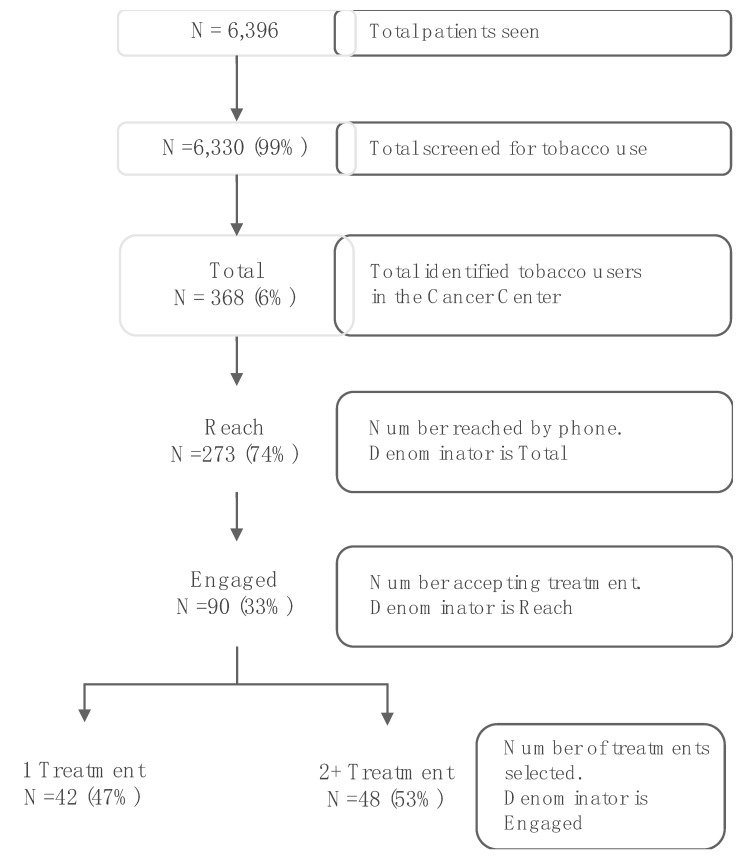
Outreach and Engagement for Tobacco Treatment Services in the QI Pilot.

The pilot launched in January 2019 in Head and Neck Oncology, followed by Thoracic Oncology and GI Surgery Oncology starting in July 2019. By December 2019, a total of 6396 patients were seen at the three clinics during this pilot phase. Of patients seen, 6330 (99%) patients were screened for current tobacco use and 368 (6%) tobacco users were identified. All identified tobacco users were opted into treatment and received phone outreach from the tobacco treatment service and information through their EHR using a secure messaging system. Shown in Figure 1, 273 (74%) patients were reached by phone and 90 patients (33% of those reached by phone) engaged in treatment, with 42 (47%) choosing either behavioral therapy or pharmacotherapy and 48 (53%) choosing combination treatment (behavioral therapy and pharmacotherapy). For the 90 patients engaged in tobacco treatment, 9 patients (10%) requested quitline e-referrals; 61 patients (68%) engaged in individual/family or group counseling services, with 15 (25%) completing 3 sessions; 59 patients (66%) received NRT, with 48 refills provided; and 24 patients (27%) received medication consultation for varenicline or bupropion.

To date, 44 patients have come up for their 6-month follow-up and 9 have reported being tobacco-free (20% of those engaged). Outcome data continue to be collected for patients at 6-months posttreatment initiation.

By 11 months, with building evidence of feasibility, acceptability, and efficacy, the tobacco treatment service with the opt-out approach was expanded to 11 additional SCC clinics, for a total of 14 clinics. Since the expansion on November 23, 2019 to February 15, 2020, 13,013 patients were screened for tobacco use and 435 patients (3%) were newly identified as tobacco users. All 435 patients were called by the tobacco treatment service within 1 week of being identified in clinic and were provided information on quitting tobacco use through their EHR. The average number of new smokers identified per week has increased from 3 to about 35, since expansion from the pilot phase. Thus far, a total of 600 patients (75%) have been reached by the team’s tobacco treatment specialist. To date, 181 patients (30% of those reached) have engaged in treatment with 108 (60%) choosing one treatment modality and 73 (40%) choosing combination treatment. Outcome assessments will continue to evaluate the efficacy of treatment service delivery for supporting long-term abstinence from tobacco use.

## 4. Discussion

As part of a national movement supported by NCI, this QI initiative aimed to integrate tobacco cessation treatment into cancer care at Stanford and to improve tobacco treatment service utilization to help patients become tobacco-free. Within 6 months of initiating the QI initiative in a single pilot clinic, two additional clinics were added. Within 11 months, an additional 11 clinics were added for a total of 14 clinics. 

In the pre-implementation assessment phase, common barriers to treatment were identified including reliance on clinician referrals, lack of insurance coverage, distance of travel to counseling sites, and fear of stigma around counseling services, which had been centered in psychiatry. Through employing the opt-out approach, offering treatment via telemedicine, and providing treatment as a covered benefit for patients and family members, this QI initiative maximized treatment access and provided individualized treatment planning. The tobacco treatment model offered a menu of treatment services to help patients meet their quit goals. Screening and treatment delivery processes were incorporated into current workflows to reduce time and costs for patients and the medical center without compromising patient–provider interpersonal contact and quality of care. To date, the quit rate is 20% at the 6-month follow-up. 

Cancer patients who smoke report higher motivation to quit—but not higher quit rates—relative to the general population [28,29]. Hence, a cancer diagnosis offers a unique and critical opportunity to help patients quit. However, given the general lack of success in the tobacco treatment research literature [22], tobacco treatment in cancer care may require new models of engagement and delivery. This QI initiative experience identified the need to provide flexible and individualized treatment interventions. Patients often feel burdened by the immediate consequences of their cancer diagnoses and treatment, which puts the consequences of long-term tobacco use in their periphery. Utilizing motivational interviewing techniques, the treatment team focused on rapport building and engagement. In addition, the initiative utilized a workflow that provided feedback and consultation with the patient’s medical team to enhance coordination of patient-centered care and to close the treatment loop. 

This QI initiative observed a level of patient tobacco treatment engagement higher than other comprehensive tobacco treatment services within oncology settings [30,31], which tend to be about 20%. With regard to abstinence outcomes, the University of Texas MD Anderson Cancer Center, which provides a comprehensive approach to tobacco treatment, has reported a 9-month abstinence rate of 38% among 2779 individuals treated from 2006–2013. Automatic referral systems that identify current tobacco users or recent quitters at clinical or preclinical evaluations have been found to dramatically increase participation in tobacco treatment in cancer care [29,30]. 

### Future Work

The tobacco treatment team anticipates expansion of the automated referral service across the institution’s cancer network, which includes 2 additional sites in Northern California. The team is investigating the use of mail and digital solutions to further extend outreach and workflow efficiencies. Anticipated is an increase in patient engagement in treatment of 8 to 10 patients per week (about 400 to 500 patients within the next year), which with the supervised trainee model and virtual pharmacy is doable. Once the process and infrastructure are developed for the cancer network, the team plans to expand services across all of Stanford Health Care. 

This practice-based QI evaluation has notable limitations. Findings may not generalize to other cancer centers. For example, the clinical capacity to call all patients identified as tobacco users may be more challenging in systems with higher smoking prevalence. Relying on self-report, tobacco use may have been underreported due to the stigma of smoking and having cancer or because of smoking being contraindicated for surgery or a cancer treatment medication [32,33,34]. The treatment model is time and resource intensive, as person-to-person connections were prioritized, and required administrative oversight as the service’s documentation and referral systems were not well embedded in the institution’s operations. Given these considerations, the scalability of the model requires further testing.

Despite these limitations, the process described here is offered as a potential model for QI initiatives to integrate tobacco cessation treatment into cancer care. Quitting smoking supports positive outcomes in the long-term care of cancer survivors [19,35]. The treatment offerings and model have engaged patients in a high touch manner of frequent person-to-person interactions, which opened opportunities to tailor and meet the individual needs of patients. 

## 5. Conclusions

The initial results of this QI tobacco treatment initiative demonstrate that providing comprehensive tobacco treatment services to patients within an oncology setting can result in higher tobacco treatment engagement. A tobacco treatment intervention implemented as a covered benefit of cancer care was found to be feasible and acceptable to clinicians and patients. Offered as evidence from practice-based QI evaluation, further testing of the model’s feasibility and scalability is warranted.
